# Hybrid Nanocomposites of Cellulose/Carbon-Nanotubes/Polyurethane with Rapidly Water Sensitive Shape Memory Effect and Strain Sensing Performance

**DOI:** 10.3390/polym11101586

**Published:** 2019-09-27

**Authors:** Guanzheng Wu, Yanjia Gu, Xiuliang Hou, Ruiqing Li, Huizhen Ke, Xueliang Xiao

**Affiliations:** 1 Key Laboratory of Eco-Textiles, Ministry of Education, Jiangnan University, Wuxi 214122, China; 18084721450@163.com (G.W.); yanjiagu1984@163.com (Y.G.); LiRuiqing_1997@163.com (R.L.); 2 Shenzhen Digital Life Institute, Shenzhen 581000, China; 3 Fujian Key Laboratory of Novel Functional Textile Fibers and Materials, Minjiang University, Fuzhou 350108, China

**Keywords:** hybrid nanocomposites, rapid recovery, sharp memory polymer, strain sensor

## Abstract

In this work, a fast water-responsive shape memory hybrid polymer based on thermoplastic polyurethane (TPU) was prepared by crosslinking with hydroxyethyl cotton cellulose nanofibers (CNF-C) and multi-walled carbon nanotubes (CNTs). The effect of CNTs content on the electrical conductivity of TPU/CNF-C/CNTs nanocomposite was investigated for the feasibility of being a strain sensor. In order to know its durability, the mechanical and water-responsive shape memory effects were studied comprehensively. The results indicated good mechanical properties and sensing performance for the TPU matrix fully crosslinked with CNF-C and CNTs. The water-induced shape fixity ratio (*R_f_*) and shape recovery ratio (*R_r_*) were 49.65% and 76.64%, respectively, indicating that the deformed composite was able to recover its original shape under a stimulus. The TPU/CNF-C/CNTs samples under their fixed and recovered shapes were tested to investigate their sensing properties, such as periodicity, frequency, and repeatability of the sensor spline under different loadings. Results indicated that the hybrid composite can sense large strains accurately for more than 10^3^ times and water-induced shape recovery can to some extent maintain the sensing accuracy after material fatigue. With such good properties, we envisage that this kind of composite may play a significant role in developing new generations of water-responsive sensors or actuators.

## 1. Introduction

In the past decades, shape memory polymers (SMPs) have gained attention because of their responsive manners to various types of stimuli (including water, electrical, heat, and pH) [1]. Visually, SMPs can recover their original shapes from temporarily deformed shapes when exposed to appropriate environmental stimuli [2,3,4], namely, the shape memory effect (SME), which is due to their unique molecular structures. According to a proposed structural model of SMP, a polymer with SME should be composed of molecular switches and netpoints [3,4,5]. When exposed to some predetermined external stimuli, the molecular switches play an important role in dominating the shape fixity and recovery, which may be crystalline phase or amorphous polymer chains. Networks may consist of either chemical or physical crosslinking points that determine the permanent shape [6]. Regarding triggers, like electric, pH, light, organic solvents, and even water [7], SMPs can be classified into many types, like temperature-actuated SMPs, electric-actuated SMPs, pH-actuated SMPs, light-actuated SMPs, solvent-actuated SMPs, and water-actuated SMPs [8,9]. In recent years, SMPs have been considered as one of the most promising smart materials, receiving extensive interest from scientific researchers [10,11].

The advantages of SMPs have resulted in great potential applications in many areas. For instance, smart fabrics, strain sensors, biomedical materials, and aerospace applications [12,13,14]. Nevertheless, some obstructions still exist that prevent SMPs’ widespread application. Customarily, in comparison with shape memory alloys (SMAs), SMPs have relatively low recovery stress [15]. This is a limiting factor in many applications, particularly where SMPs must conquer a large resisting stress during shape recovery [16]. Therefore, in order to enhance the mechanical properties and to obtain multiple functionalities, many kinds of nano-fillers have been used to modify polyurethane [17,18]. 

Zhu et al. [19] reported a water-actuated SMP with an unprecedentedly rapid switchable SME that was fabricated by adding a crystalline nano-whiskers percolation network to TPU. Li et al. [20] developed pH-actuated SMP nanocomposites by blending poly(ethylene glycol, PEG)-poly(ε-caprolactone, PCL)-based polyurethane with cellulose nanocrystals (CNCs), which were functionalized with pyridine moieties (CNCs-C_6_H_4_NO_2_) through hydroxyl substitution of CNCs with pyridine-4-carbonyl chloride and carboxyl groups (CNCs−CO_2_H) via 2,2,6,6-tetramethyl-1-piperidinyloxy (TEMPO)-mediated surface oxidation. Liu et al. [21] introduced CNC into the mixture of PEG and PCL. The mechanical properties of the mixture were significantly improved, and a nanocomposite SMP that was simultaneously thermal- and water-responsive was thereafter prepared. Through these previous studies, we found that CNCs have a good reinforcing effect on the mechanical properties of the matrix of SMP. Moreover, due to the high strength, Young’s modulus, and conductivity of carbon nanotubes [22,23], such nanomaterials can enhance the matrix functions of SMPs. Yu et al. [24] reported that a small proportion of CNTs can improve the thermal properties of SMP nanocomposites. Du et al. [25] developed an electroactive SMP that was prepared by PVA and CNTs. It was reported that the reinforcement of multi-walled CNTs can lead to enhanced electrical and thermal characteristics of the PVA matrix. Chen et al. [26] developed CNTs array-based epoxy-SMP (ESMP) nanocomposites. In this material, the CNTs were interconnected with each other and a three-dimensional (3D) network was formed. As a result, both the thermal and electrical conductivities of the high array of CNTs/ESMP nanocomposites were higher than the random CNTs/ESMP composite fabricated by conventional high-speed mechanical stirring [27].

Further, sensors, as one of the most useful applications of SMPs, plays an important role in human motion monitoring. A series of important achievements have been made in the field of flexible wearability for scientific researchers [28,29,30]. For example, Zhou et al. [31] developed a strain sensor with high sensitivity, high tensile property, and high linearity, which was fabricated by wet spinning of CNT-doped thermoplastic elastomer. Therefore, the mechanical properties of SMPs may be highly enhanced by CNCs, and the electrical conductivity and thermal conductivity of SMPs may be given by CNTs [32]. In this work, a multi-responsive SMP composite based on TPU was fabricated by crosslinking with CNCs and CNTs, hopefully in combination with the advantages of CNCs, CNTs, and TPU for fine mechanical properties, stimuli-responsive, and electrical conductivity, in order to obtain an ideal sensing performance.

## 2. Materials and Methods

### 2.1. Raw Materials

Thermoplastic polyurethane granules (TPU, type of BT-70ARYU, hardness of 70 ± 2 Shore A, density of 1.13 g/cm^3^) were purchased from Shanghai Yongyi Industrial Co., Ltd. (Shanghai, China). Mn ≈ 0.2 × 10^5^. Cotton cellulose nanofiber (CNF-C, with a large amount of CNCs, solid content is 1.0 wt%, particle size is around 6.5 nm, length is around 1000 nm) was purchased from Guilin Qihong Technology Co., Ltd. Carbon nanotubes size (CNTs, content is 5.0 ± 0.2%, diameter is in the range of 30 and 60 nm, length is 10 to 30 μm) were purchased from Shenzhen Tuling Evolution Technology Co., Ltd. (Shenzhen, China) N, N-dimethylformamide (DMF, CP, ≥99.0%) was purchased from Sinopharm Chemical Reagent Co., Ltd. The linear type of TPU was applied without further purification, but CNF-C and CNTs were freeze-dried for 72 h, and CNTs were ground into powder. Then the raw materials were kept in a dry atmosphere for further use.

### 2.2. Preparation of TPU/CNF-C/CNTs Hybrid Films

TPU/CNF-C/CNTs hybrid films were prepared through solution blending [33]. Originally, the TPU/CNF-C solution and CNTs solution were prepared separately. Afterwards, the two kinds of solutions were mixed together. Taking the TPU/CNF-C/CNTs (100:5:5 wt%) (named TPU/CNF-C/CNT-5) as an example, the specific steps were as follows: Firstly, TPU (4.5 g) and CNF-C (0.225 g) were dissolved in 25 mL of DMF solvent at 75 °C for 1 h with the aid of magnetic stirring. CNTs (0.225 g) nanoparticles were dispersed in 20 mL of DMF solvent for about 0.5 h under ultrasonic conditions. Afterwards, the CNTs solution was gradually poured into the TPU/CNF-C solution, then the mixture was further stirred for 2 h. Ultimately, the TPU/CNF-C/CNTs mixed solution was poured into a petri dish and preserved in an oven at 75 °C for enough time to eliminate the solvent inside, then a TPU/CNF-C/CNTs film was obtained. In order to investigate the effect of CNTs on the properties of the TPU/CNF-C/CNTs system, a series of nanocomposite thin films were prepared with different contents of CNTs, as shown in Table 1. Here, “CNT-X” followed by a number means the approximate weight percentage of CNTs in the fabricated system.

For the sake of measuring properties of as-made films, all samples were processed into the same thickness of 0.15 mm under the melt temperature of 130 °C and the compression pressure of 15 MPa (R-3212 type of hot press, Wuhan Qien Sci-Tech Co., Ltd., China). Then, the TPU/CNF-C/CNTs thin films with the same thickness were obtained using the hot-press process [34].

### 2.3. Characterization by Attenuated Total Reflectance Fourier Transform Infrared Spectroscopy (ATR FT-IR)

Fourier transform infrared spectroscopy spectra of the as-made samples were analyzed using a Nicolet is 10 spectrometer (Thermo Fisher SCIENTIFIC-CN, Waltham, USA) in the ATR module at room temperature from 4000 to 560 cm^−1^ at a resolution of 4 cm^−1^.

### 2.4. Characterization by Wide Angle X-Ray Diffraction (WAXRD)

Wide angle X-ray diffraction (WAXRD) is an X-ray diffraction technique that can be used to determine the crystalline structure of polymers [35]. The crystalline structure of samples was investigated using a D2 PHASER X-ray powder diffractometer (Brook AXS Ltd., Karlsruhe, Germany). The X-ray source was the ceramic type, Cu target, and the power of the optical tube was 2.2 kW. The range of the scanning angle was 5 to 90°, resolution ratio was 0.026, and the scanning rate was 2°/min.

### 2.5. Characterization by Differential Scanning Calorimetry (DSC)

The thermal properties of samples were investigated using a differential scanning calorimeter (DSC) Q200 (TA Instruments-Waters LLC, Hangzhou, China), which can be used to characterize the glass transition temperature [36]. A sample of about 5 mg, which was cut from the compression-molded sheet, was directly heated from 40 to 120 °C at a heating rate of 10 °C/min. All the measurements were conducted in a nitrogen (N_2_) atmosphere.

### 2.6. Characterization by Tensile Tests

The tensile tests were carried out at room temperature (25 °C) using a CMT4101 Electronic Universal Testing Machine (Shenzhen New Sansi Material Testing Co., Ltd., Shenzhen, China) at a constant crosshead rate (30 mm/min). The rectangular film sample for the tensile tests was cut from the hot-pressed sheet with a width of 10 mm and a length of 60 mm. The original distance between two clamps was set at 50 mm [37]. All samples were stretched until breakage for a single tensile test.

### 2.7. Shape Memory Behavior Measurements

**Figure 1:**
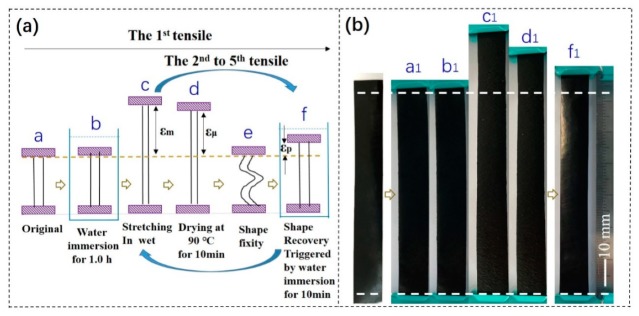
The cyclic tensile test to demonstrate the program of water-stimulus SME.

Different from the single tensile test, to study the strength and strain at break, a cyclic tensile program was carried out to investigate the shape memory effect of the as-made samples, as shown in Figure 1a. Here, the sample was firstly immersed in deionized water (set at 25 °C) for 1 h (Figure 1a–b). Then, the sample was taken out quickly and stretched at a constant cross-head rate (30 mm/min) to a strain ($\varepsilon_{m}$) of 40% at an ambient temperature of 22 °C (Figure 1a–c). Then, the stretched strain was maintained for 10 min (Figure 1a–d), and the sample was dried by an air blower during the strain. A fixed strain ($\varepsilon_{u}$) was recorded accordingly. Subsequently, the stretched film was released to the original clamped distance with the temporarily fixed strain (Figure 1a–e), and the sample was immersed in water again for 10 min of shape recovery (Figure 1a–f). The second tensile started from the recovered shape (a residual/unrecovered strain *ε_p_*) of the sample. The operations were repeated three times to form a water-responsive cyclic tensile test for SME quantification. The cyclic tensile test followed Zhu et al.’s work [19].

In Figure 1a, N is the cycling number, *ε_m_* is the set strain for shape fixation, $\varepsilon_{u-N}$ means the real fixed strain at the step of Figure 1a–d, and $\varepsilon_{p-N}$ means the unrecovered strain after the shape recovery. The shape fixity ratio (*R_f_*) and shape recovery ratio (*R_r_*) were calculated according to Equations (1) and (2) [38,39]:

(1) $$R_{f}=\frac{\varepsilon_{u-N}}{\varepsilon_{m}}\times 100\%$$

(2) $$R_{r}=\frac{\varepsilon_{m}-\varepsilon_{p-N}}{\varepsilon_{m}-\varepsilon_{p-\left(N-1\right)}}\times 100\%$$

Figure 1b is the real sample in the SME test diagram corresponding to the schematic of Figure 1a. From Figure 1b (a_1_ to f_1_), the illustration shows the process of shape fixity and shape recovery in SME.

### 2.8. Tests of Sensing Performance

The sensing performance tests were also carried out at room temperature (20 °C) using the CMT4101 Electronic Universal Testing Machine (Shenzhen New Sansi Material Testing Co., Ltd., Shenzhen, China) [37]. The rectangular film sample for the tensile tests was cut from the hot-pressed sheet with a width of 10 mm and a length of 50 mm. The distance between two clamps was maintained at 40 mm, then the resistance of the sample was tested by a multimeter during the stretching. The two ends of the sample were fixed on the fixture of the multimeter. The resistance of the corresponding point was recorded at a frequency of 1 Hz, and the stretched strain was set as 40%.

## 3. Results and Discussions

### 3.1. Physical Properties of TPU/CNF-C/CNTs Films

**Figure 2:**
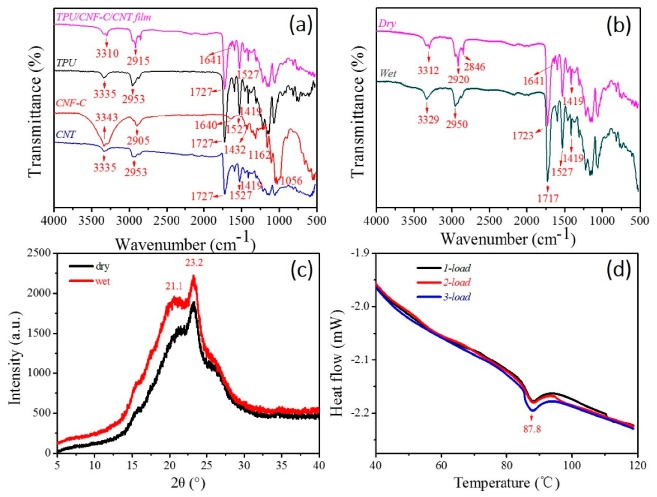
Chemical structure and physical properties of as-made water-responsive polyurethane composition, (**a**) FT-IR results of TPU/CNF-C/CNTs film, TPU, CNF-C, CNTs; (**b**) FT-IR results of TPU/CNF-C/CNTs films under original (dry) and water (wet); (**c**) XRD patterns of TPU/CNF-C/CNTs film under dry and wet states; (**d**) DSC characterization of TPU/CNF-C/CNTs film from 40 to 120 °C for three loads.

The chemical structures and physical properties of the as-made water-responsive polyurethane nanocomposites were characterized by FT-IR, XRD, and DSC, respectively, as shown in their spectra in Figure 2.

**Figure 3:**
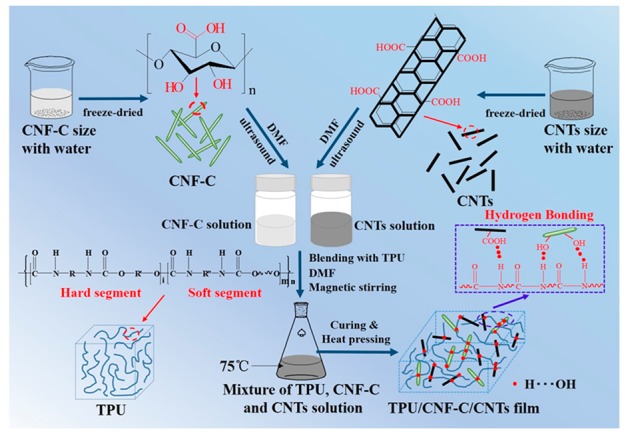
Schematic illustration of the hydrogen bonding interaction mechanism among molecules of TPU, CNF-C, and CNTs, and the film formation of TPU/CNF-C/CNTs.

Among them, FT-IR was adopted to characterize the chemical interaction of molecules of TPU, CNF-C, and CNTs, and the change of TPU/CNF-C/CNTs from dry to wet states. Specifically, in Figure 2a, the characteristic peaks of –NH at 3335 and 2953 cm^−1^ were slightly interrupted by the similar polar group of –OH from CNF-C and CNTs. The unique characteristic peak, representing the hydrogen bonding interaction of –COOH and –OH, at 1640 cm^−1^ from CNF-C was also brought into the composite system by the observed peak at 1641 cm^−1^. The other characteristic peaks were the main peak features from TPU. As shown in Figure 2b, the penetration of aqueous molecules into the composite system breaks up the hydrogen bonding of –COOH and –OH, giving rise to the disappearance of the characteristic peak at 1641 cm^−1^ for the wet samples [40]. Because of hydrophobic nature of the TPU matrix and the ATR FT-IR characteristic method (10 μm of IR depth into the sample surface), the wet sample of the nanocomposite displays a slightly broad peak at 3400 cm^−1^ close to 3329 cm^−1^ due to water volatilization. In turn, a drying process eliminates the aqueous molecules, with a disappearance of the characteristic peak of the –OH group (3400 cm^−1^), re-forming the hydrogen bonds and showing a characteristic peak at 1641 cm^−1^. This reversible behavior related to the conversion between dry and wet conditions suggests that the intermolecular hydrogen bonds undergo reversible destruction and formation processes accordingly. Combining the results of the FT-IR tests, a scheme of the interfacial interactions mechanism based on hydrogen bonding is exhibited in Figure 3.

The nanoparticles of CNF-C and CNTs were derived from the CNF-C and CNTs size with water via a freeze-dried process. Dissolved TPU solution was blended evenly with CNF-C and CNTs nanaoparticles, then TPU/CNF-C/CNTs nanocomposite films were obtained after a curing process (75 °C). Furthermore, a large amount of –OH and the rigid structure of the CNF-C contributed to dual functions of physical and chemical crosslinking points in the nanocomposite film. A great quantity of the –COOH group was distributed on the surface of rod-like CNTs, which could perform a similar function as CNF-C. In addition, CNF-C and CNTs can be combined via hydrogen bonding. After blending, CNF-C and CNTs can disperse well in the TPU matrix. The existence of the hydrogen bonding among the TPU matrix, CNF-C, and CNTs can form a three-dimensional network structure [34].

The XRD patterns of TPU/CNF-C/CNTs hybrid films were obtained under two states as shown in Figure 2c. The diffraction shoulders and peaks at the dry state were due to the wetting and hydration with similar shape peaks, especially at the abscissa of 2θ = 21.1° and 23.2°, indicating the strong characteristic β-keratin crystalline phases. The intensities of both characteristic peaks at the wet state were greater than those of the dry state. This indicates the invariable amount of the crystalline phase during water-stimuli SME. The existing XRD peaks of TPU/CNF-C/CNTs under dry and wet conditions indicate that the crystalline phase may take a net point role in the water-sensitive SME of the hybrid system.

The DSC patterns of TPU/CNF-C/CNTs hybrid film were obtained from 40 to 120 °C under three heating loads, as shown in Figure 2d. The DSC curves coincide with each other basically. Each curve manifests that the enthalpy decreases during the heating process of the film, and reverses its initial value during the cooling process, which is in relation to the films in the progress of shape recovery. The endothermic peak indicates that the glass transition temperature (Tg) of as-made film sample is 87.8 °C. The measured Tg provides a theoretical basis for setting the shape fixation temperature, which should be a value higher than Tg. In this work, a temperature of 90 °C was used to set the temporary shape fixation of sample.

### 3.2. Mechanical Properties of TPU/CNF-C/CNTs Films

**Figure 4:**
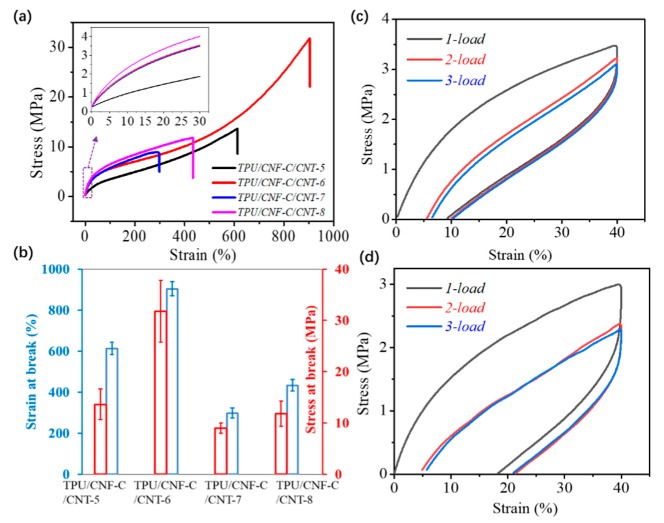
Measured mechanical properties of nanocomposite films, (**a**) typical tensile stress–strain curves, the inset shows the stress–strain curve of each sample stretching up to 30%; (**b**) the strain and stress values at break for TPU/CNF-C/CNTs-5, TPU/CNF-C/CNTs-6, TPU/CNF-C/CNTs-7, TPU/CNF-C/CNTs-8; (**c**) experimental cyclic tensile of TPU/CNF-C/ CNTs-6 film without using any stimulus; (**d**) cyclic tensile of TPU/CNF-C/CNTs-6 film under water stimulus.

The measured tensile stress–strain curves are shown in Figure 4a for as-made TPU/CNF-C/CNTs-5, TPU/CNF-C/CNTs-6, TPU/CNF-C/CNTs-7, and TPU/CNF-C/CNTs-8. Each film sample presents a smoothly nonlinear elastic behavior until the breaking point, which is mainly ascribed to the fracture of the film along the stretching direction. Some studies, including the new reports [41,42,43,44], suggest that the commixture with CNF-C and CNTs can enhance the mechanical properties of TPU significantly, especially the tensile strength and elongation at break (Figure 4b), but with the continued increase of the content of nanoparticles, the strength of TPU/CNF-C/CNTs nanocomposite films would decrease slowly. Figure 4b shows the best mechanical properties of the TPU/CNF-C/CNTs-6 film. It can achieve 31.78 MPa, and the corresponding strain at break is 904.1%. In view of the above mechanical performance, the sample of TPU/CNF-C/CNTs-6 was selected for the tests of typical SME and sensing performance [44].

For the measured cyclic tensile curves of Figure 4c,d, Equations (1) and (2) can be used to quantitatively analyze the SME of film sample responsive to water. Figure 4c indicates a fixed strain *R_f_* of measured 25.27% $\varepsilon_{u-N}$ = 10.1%, $\varepsilon_{m}$ = 40%) while Figure 4d presents the fixed strain, *R_f_*, measured as 49.65% $\varepsilon_{u-N}$ = 19.50%, $\varepsilon_{m}$ = 40%). This indicates that water is an effective stimulus to fix the temporary shape of the as-made nanocomposite film.

In Figure 4a, the inset image shows a slightly nonlinear increase relationship of stress and strain until 30%, and reflects a unique tensile deformation of the macromolecular sample before yield point. Plastic deformation of the film was increased after the yield point, which gives the possibility of shape fixation and recovery for a specific stimulus. Here, for *R_r_*, water as a kind of stimulus leads to a larger degree of shape recovery (76.64%) than the film without stimulus (45.54%). In this case, water initiates a higher recovery by the stress released from net points via the switch units (like hydrogen bonds), which are unlocked through aqueous molecules. To illustrate the point, all the tests were conducted under the same tensile speed in 30 mm/min, and remained at the extreme point for 10 min [45]. Contrasting findings showed that water can improve the *R_f_* and *R_r_* values of as-made composite samples effectively. The values depend on the interaction of the stimulus, net points, and switches [45]. In such a water-sensitive SMP, CNF-C and CNTs were considered as net points, and the mechanical properties of the nanocomposite SMP were significantly enhanced. Through FT-IR characterization, the intermolecular hydrogen bonding was regarded as the corresponding switch. The combination of a percolation network CNF-C, CNTs, and TPU matrix is the microstructural precondition for rapid switchable water-sensitive SME in TPU/CNF-C/CNTs films. Original TPU/CNF-C/CNTs film can be softened via water molecules, with the breakage of hydrogen bonds among the TPU and nanoparticles. The manually deformed shape was relatively easy to be fixed because a drying process can lead to shape fixation through the formation of a three-dimensional network, with individual nanoparticles crosslinking by hydrogen bonds after the removal of the water molecules. In the procedure of shape recovery, water as an external stimulus leads to an opening of switches among the TPU and nanoparticles, and a shape recovery would be noted by the water triggering the system [19].

### 3.3. Rapid Response to Water Stimulation

**Figure 5:**
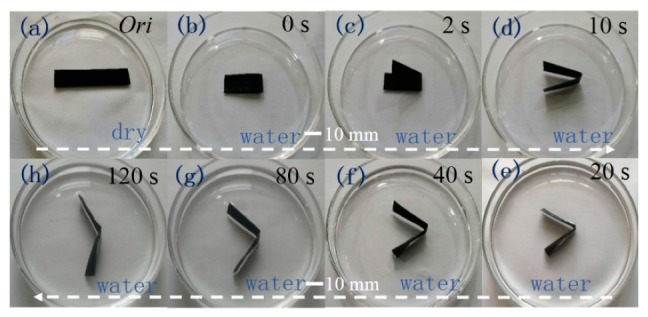
Shape recovery of manually deformed TPU/CNF-C/CNTs film in water.

Figure 5 shows the process of TPU/CNFC/CNTs film under a continuity of water stimuli to investigate its SME. First, a dry straight film was folded in half, and then was immersed in water (20 °C) for one hour. The folded film was then taken out of the water under constrained conditions and heated in an oven to fix the folded shape at 90 °C. The dried folded shape was then fixed, as mentioned above, and the as-made film with structural water and hydrogen bonds was fixed with the shape. The folded film was then put into water (20 °C), and a response of the film was noted by recording the shape recovery behavior, as shown by the few screenshots of the whole recovery process in Figure 5.

With an increased time of the temporarily fixed shape of film in water, the folded angle of the sample increased gradually. As shown in Figure 5b–h, a turning over behavior of the film in water is noted at step b–d, indicating enough stress was released from the switch opening by water even to the extent that it was higher than the film’s weight. Under deep impregnation of water into the film, the final recovered shape is close to its original shape at 120 s, indicating a relatively fast response of the as-made film with manually deformed shape exposure to water. Then, for such a hybrid nanocomposite, aqueous molecules play an important role in water-sensitive SME, and hydrogen bonds ought to be the switch unit in the system [46]. The shape-fixed film quickly recovers its original state because of the quick action of aqueous molecules and switch units.

### 3.4. Characterization of Sensing Performance

**Figure 6:**
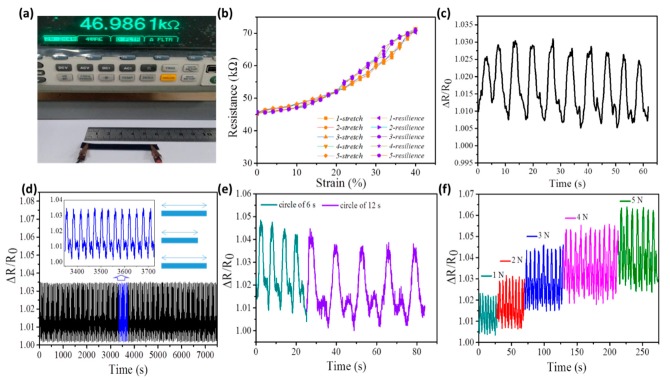
Characteristics of as-made TPU/CNF-C/CNTs nanocomposite as strain sensor, (**a**) electrical conductivity of TPU/CNF-C/CNTs film; (**b**) cycling test showing relative resistance change with tensile strain in the range of 0% to 40%; (**c**) periodicity test curves of the film sample under a constant stress of 1.33 MPa; (**d**) repeatability test curves under a constant stress of 2.0 MPa for the change of ∆R/R_0_ values after 1000 cycles of repeatable stretch, inset: enlarged image of 3348 to 3742 s; (**e**) relative resistance change of strain sensors during the cyclic stretching with two different stretching rates (30%, 60%); (**f**) repeatability test curves under constant stresses of 0.67, 1.33, 2.0, 2.67, and 3.33 MPa for the change of ∆R/R_0_ values.

With the involvement of CNTs in composites, electrical conductivity was applied to the film. Elasticity and electrical conductivity endow the developed nanocomposite with a sensing function. However, for most strain sensors in composite systems, strain relaxation is an inevitable factor, leading to sensing failure for thousands of usages, thus SME may be an effective way to enhance the sensing durability. Under this consideration, the factors affecting a sensor performance were investigated with the involvement of water-sensitive SME. Here, in Figure 6a, electrical conductivity was tested by an electrochemical workstation. The measured resistance for an original-shaped sample was 46.986 kΩ (the length, width, and thickness of the sample were 100, 10, and 0.15 mm) with a calculated electrical conductivity of 0.142 S/m.

**Figure 7:**
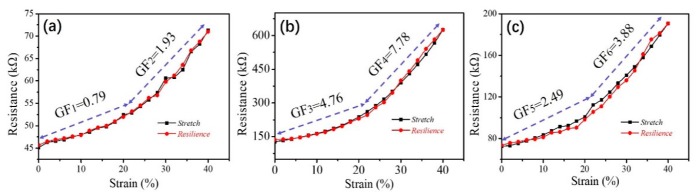
Cyclic test of relative resistance change as strain increases up to 40%, (**a**) origin sample; (**b**) temporarily stretched sample (strain of 40%); (**c**) recovered sample from the fixed strain of 40%.

The strain–sensitivity relationship was measured by an ALJ-50HB manual screw tension tester, which has an electrochemical workstation to record the resistance (Figure 6b). It expounds the change of the relative resistance (∆R/R_0_) of the sensor related to the tensile strain (ε) of the film, which is defined as the ratio of resistance change (∆R) to the original resistance (R_0_) before stretching [47]. Here, a monotonic increase of ∆R/R_0_ can be seen with the strain between 0% and 40% (five times reversion). The increase of tensile strain leads to a gradual increase of non-communicating CNTs particles in the film, leading to an increase of the resistance. Gauge factor (GF, defined as (∆R/R_0_)/ε) was reflected by the slope of the relative resistance change versus the applied strain. These resistive-type strain sensors rely on numerous features, such as the content of conductive components in the sensor. Then, the curve can be divided into two phases, 0% to 20% and 20% to 40% string with a GF of 0.79 and 1.93, respectively, which will be discussed in Figure 7 in detail.

Besides the sensitivity study, the periodicity, signal stability, and repeatability are all parameters in consideration of the new composite film for the suitability of a strain sensor [48]. Here, Figure 6c presents a stable periodicity and rapid response during the stretch–release tests under a regular intermittent stress of 1.33 MPa for applied strain of 20%. Each periodic stretch–release for 20% of strain was completed in 6 s corresponding to nearly 1.03 of ∆R/R_0_. Moreover, the sensing stability was measured for the strain sensor under a stress of 2.0 MPa like the periodicity test. The change of ∆R/R_0_ values under 20% of the applied strain was repeated for 1000 cycles, as shown by the measured broken lines in Figure 6d, indicating a good signal repeatability using the as-fabricated composite film.

As shown in Figure 6b, a stretching curve with the relationship of resistance and strain gives two different applied strains (20%, 40%) for the change of ∆R/R_0_. Figure 6e gives the signal periodicity (in 6 and 12 s) of cyclic applied strain, indicating the strain sensor had a high responsiveness of resistance (1.05 = ∆R/R_0_) in small applied strains. The developed strain sensor manifests high reliability and reversible resistance change. Then, under different applied stresses of 0.67, 1.33, 2.0, 2.67, and 3.33 MPa for the variation of ∆R/R_0_, the strain sensor presents the corresponding strains from 10% to 60%, as shown in Figure 6f, related to the variation of ∆R/R_0_ from 1.025 to 1.065. Here, it is noted that a footstep incremental curve appears on the repeatability test with the increase of applied stress, which is ascribed to the limited recovery time to the applied strain, giving rise to a gradual increase of the initial value of ∆R/R_0_ [49].

### 3.5. Comparison of Sensing Performance Before, During, and After SME

Figure 7 shows the change of the measured electrical resistance of the sample with the increase of strain up to 40%, in which three kinds of sample dry states are illustrated based on a water-induced SME program, including original, temporarily fixed, and recovered shapes of nanocomposite samples.

In Figure 7a, the length, width, and thickness of the measured sample are 50, 10, and 0.15 mm, respectively. The tested resistance–strain curve show two phases of GF values of 0.79 and 1.93, indicating the sensitivity of the sample sensor increases with the increase of the tensile strain, and the non-linear growth of the slope also conforms to the tensile properties of polymer materials. Figure 7b shows the highly increased resistance of the sample with a continued increase of the stretch strain beginning from its temporarily fixed strain. The stretch curve shows two phases roughly (0%–20% and 20%–40%) with GF mean values of 4.76 and 7.78, respectively, which are larger than the values from Figure 7a. This indicates the sample has a higher sensitivity (higher GF values) in a temporarily stretched shape, because the average distance of two neighboring CNTs in microscale is enlarged during stretch. This leads to a smaller number of contacts of CNTs in the fabricated nanocomposite and an increased resistance [47]. The data in Figure 7c is derived from the sensitivity of the recovered dry sample induced by water. Here, the measured GF values are 2.49 and 3.88 for two phases of tensile strains.

In comparison with Figure 7a,c, it must be pointed out that the original and recovered samples present different sensing sensitivities. An indication is explored for a reason that the distribution of CNTs in the recovered sample has an irreversible destruction even after a shape recovery process. Nonetheless, the recovery process still reduces GF values from shape fixed states, indicating a repair function to the sensor, especially when it experiences long-time usage with relaxation. Thus, the sensor with SME may satisfy many application scenarios around water/wet stimulation by exploring the sensor’s sensing performance in three situations: In-situ, stretched and fixed or restored, and recovered states [47].

### 3.6. Application Demonstration of Limb Motion

**Figure 8:**
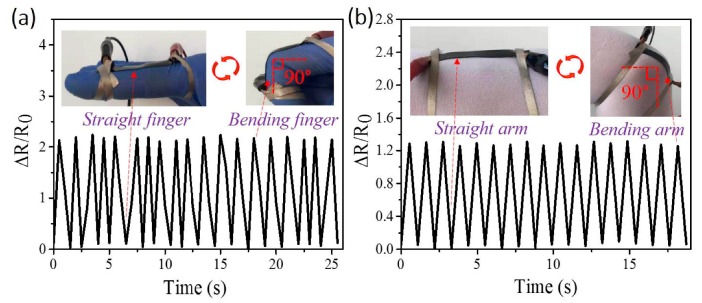
Human motion monitoring using the as-fabricated sensor, (**a**) relative resistance variations (ΔR/R_0_) versus time for blending and release of the index finger, inset: snapshots of one bending cycle. (**b**) ΔR/R_0_ for TPU/CNF-C/CNTs strain sensors according to bending motions, inset: one bending cycle of arm, the bending motion degree of the arm at angle of 90°.

The as-made TPU/CNF-C/CNTs film strain sensors can be used for wearable electronics, because the stretchable film can easily adhere to the surface of cotton-blended spandex fabric. For human motions, different parts of the human body in diverse small scales can be detected using the strain sensors, such as movements of the finger and arm [50]. Here, the strain sensor was adhered to the joint of the index finger and the joint of the arm with cotton-blended spandex fabric using double-sided tape (inset of Figure 8a). The attached sensor recorded each bending cycle (bending finger and arm angle of 90°), with the sensor stretching and releasing once (inset of Figure 8a,b). The measured broken lines with variable ΔR/R_0_ values are related to the stretching/releasing of the length of the strain sensor via the human joints bending. The periodic sensing signals cyclically increase and decrease under variable strains as a response to the motion of different parts of the human body [51,52]. The test results showed that the strain sensor satisfies the detection requirements of finger and arm motions, and the results have reasonable accuracy to reflect each human motion. 

## 4. Conclusions

A TPU/CNF-C/CNTs hybrid nanocomposite was successful prepared with water-sensitive SME, in which the crystalline phase was characterized as net points and hydrogen bonds as the switch unit. The mechanism indicated that CNF-C brought a large amount of hydrogen bonds (FT-IR spectra) and crystals (XRD spectra) into the TPU matrix and CNTs brought electrical conductivity (multimeter test) and sensing performance into the TPU system. Experimental measurements showed that 6wt% content of CNTs in the TPU/CNF-C/CNTs sample displayed the best mechanical properties, such as the highest breaking strength and elongation. The detailed stress at break was measured up to 31.78 MPa, and the corresponding elongation was 904.09%. The combination of mechanical and conductive adaptability of a percolation network of CNF-C and CNTs, and the entropic elasticity of TPU are the basis to achieve shape fixity for temporary deformation in dry and water states, and recover the original shape after wetting, cool-off, etc. Temporary shape fixation (49.65% of *R_f_*) and shape recovery (76.64% of *R_r_*) of TPU/CNF-C/CNTs sample stimulated by water demonstrated that the as-fabricated TPU-based composite are a kind of smart and functional material.

A SME program showed that the as-fabricated TPU/CNF-C/CNTs sample displayed a fast response (120 s) of the manually deformed shape to recover its original shape when exposed to water, indicating that the involvement of CNF-C in the TPU macromolecular networks improves the water stimulus SME behavior. Furthermore, the involvement of CNTs into TPU macromolecular networks showed a good conductivity with 0.142 S/m, such that the elasticity and conductivity endowed the materials with good sensing property. The sensitivity (GF = 1.93), periodicity (10 times per minute), and signal repeatability (effective >1000) were all measured with good characteristics for applications as a strain sensor. Demonstrations of the sensor used on the bending of the finger and arm were carried out, revealing that such a material sensor can detect human motion effectively. In this work, water-sensitive SME was shown to endow the sensor with more stability when usage repeatability exceeds a point in a wet environment. The combination of SME and sensing property may apply the new material to develop next generations of water-responsive sensors, actuators, or biomedical devices.

## Figures and Tables

**Table 1:** Composition of the control sample and as-fabricated TPU/CNF-C/CNTs nanocomposites.

Sample Code	TPU/g	CNF-C/g	CNTs/g	CNTs/wt%
TPU/CNF-C/CNT-5	4.5	0.225	0.225	5.0
TPU/CNF-C/CNT-6	4.5	0.225	0.27	6.0
TPU/CNF-C/CNT-7	4.5	0.225	0.315	7.0
TPU/CNF-C/CNT-8	4.5	0.225	0.36	8.0
